# Monitoring the ventilation of living spaces to assess the risk of airborne transmission of infection using a novel Pocket CO_2_ Logger to track carbon dioxide concentrations in Tokyo

**DOI:** 10.1371/journal.pone.0303790

**Published:** 2024-05-23

**Authors:** Yo Ishigaki, Shinji Yokogawa

**Affiliations:** 1 Research Center for Realizing Sustainable Societies, The University of Electro-Communications, Chofu, Tokyo, Japan; 2 Info-Powered Energy System Research Center (iPERC), The University of Electro-Communications, Chofu, Tokyo, Japan; Satyawati College, University of Delhi, INDIA

## Abstract

We employed carbon dioxide (CO_2_) concentration monitoring using mobile devices to identify location-specific risks for airborne infection transmission. We lent a newly developed, portable Pocket CO_2_ Logger to 10 participants, to be carried at all times, for an average of 8 days. The participants recorded their location at any given time as cinema, gym, hall, home, hospital, other indoors, other outgoings, pub, restaurant, university, store, transportation, or workplace. Generalized linear mixed model was used for statistical analysis, with the objective variable set to the logarithm of CO_2_ concentration. Analysis was performed by assigning participant identification as the random effect and location as the fixed effect. The data were collected per participant (seven males, four females), resulting in a total of 12,253 records. Statistical analysis identified three relatively poorly ventilated locations (median values > 1,000 ppm) that contributed significantly (*p* < 0.0001) to CO_2_ concentrations: homes (1,316 ppm), halls (1,173 ppm), and gyms (1005ppm). In contrast, two locations were identified to contribute significantly (*p* < 0.0001) to CO_2_ concentrations but had relatively low average values (<1,000 ppm): workplaces (705 ppm) and stores (620 ppm). The Pocket CO_2_ Logger can be used to visualize airborne infectious transmission risk by location to help guide recommendation regarding infectious disease policies, such as restrictions on human flow and ventilation measures and guidelines. In the future, large-scale surveys are expected to utilize the global positioning system, Wi-Fi, or Bluetooth of an individual’s smartphone to improve ease and accuracy.

## Introduction

The ability to measure airborne bacteria and viruses in real time could serve as a crucial tool in alerting individuals to potential airborne infection transmission, thereby prompting behavior modifications to mitigate risks. While biosensors have been proposed for this purpose [1], current models are not sufficiently compact, lightweight, or cost-effective. Hence, we developed a Pocket Carbon Dioxide (CO_2_) Logger, a portable recording device with a built-in CO_2_ sensor, and conducted a field experiment on city sensing by attaching it to participants in Tokyo, Japan. We aimed to visualize areas with poor ventilation and, therefore, a high risk of airborne transmission, by analyzing data on indoor CO_2_ concentrations using citizen sensing.

Exhaled human breath contains CO_2_ concentrations of approximately 30,000 ppm, which is two orders of magnitude higher than the atmospheric level of 400 ppm, thereby, making it a reliable tracer gas to indicate indoor ventilation capacity [2, 3]. Furthermore, the exhaled breath of infected individuals contains droplet nuclei containing viruses and high concentrations of CO_2_, both of which have spreading and translocation properties. Hence, CO_2_ concentrations can be regarded as a proxy indicator of aerosol risks [4]. Several developed countries recommend maintaining CO_2_ concentrations below 800 ppm as a special exception in pandemic situations [5] as CO_2_ concentrations are recognized as an indicator of indoor ventilation and airborne transmission.

In Japan, control of indoor CO_2_ concentrations has been proposed since 1902 in the Meiji period [6]. Today, Japan’s Building Standards Act (revised and enforced in 2003) requires the installation of 24-h ventilation systems in all living rooms to prevent sick house syndrome. Moreover, it requires a ventilation capacity of at least 0.5 times/h in the living rooms of residential buildings. A ventilation capacity of 0.5 times/h is, for example, the ventilation capacity that would result in a steady-state CO_2_ concentration of 800 ppm when two people are in a room, 1,000 ppm when three people are in a room, and approximately 1,200 ppm when four people with standard activity levels are present in a 70 m^2^ space, which is the minimum standard for infection control. Maintaining CO_2_ concentrations consistently below 1000 ppm proves challenging for an average household [7]. Conversely, the Act on Maintenance of Sanitation in Buildings (enforced in 1970), which specifies environmental sanitation requirements for buildings used and occupied by a large number of people, sets standards for the control of CO_2_ concentrations across various types of buildings including entertainment venues, department stores, offices, and schools, where the area of the portion used for these specified purposes is ≥ 3,000 m^2^ (≥8,000 m^2^ for schools) (defined as specified buildings), the CO_2_ concentration is regulated at ≤1,000 ppm and periodic inspections are conducted [8].

Du et al. reported that improving the ventilation system in a poorly ventilated university building, where a tuberculosis (TB) outbreak (27 patients with TB and 1,665 contacts) occurred, and reducing the maximum CO_2_ concentration from 3,204 ± 50 to 591–603 ppm would reduce the rate of secondary infection among new contacts to zero (average follow-up period: 5.9 years); moreover, controlling CO_2_ concentrations below 1,000 ppm would reduce the incidence of TB in contacts by 97% (95% confidence interval (CI):50–99.9%) [9]. Multiple studies have reported airborne transmission of coronavirus disease (COVID-19), particularly in poorly ventilated indoor spaces [10–15]; hence, the application of CO_2_ concentration as an indicator of risk for the airborne transmission of severe acute respiratory syndrome coronavirus 2 (SARS-CoV-2) has also been proposed [16, 17].

The Wells–Riley Eq (1) is the classic model for quantitatively evaluating the risk of airborne infections [18, 19].

$$p=1-\exp\left[-\frac{\mathrm{Iqpt}}{Q}\{1-\frac{V}{Q\theta }[1-\exp\left(-\frac{Q\theta }{V})\right]\}\right]$$ (1)

Where *P* is the probability that an infected person will be infected, *I* is the number of infected persons in a closed space, *p* is the volume of breath per person (㎥/h), *q* is the rate of infectious droplets (/h) (where the *q* in a person infected with SARS-CoV-2 will be ≥100 pieces/h [20]), *t* is the time stayed by the infected person (h), *θ* is the time spent by the infected person (h), *V* is the room volume (m^3^), and *Q* is the ventilation rate (m^3^/h). The Wells–Riley equation models the probability of exposure due to a person breathing in infectious droplets emitted by an infected person as a risk quantity. Eq (1) explains that increasing *Q*, i.e., improving ventilation, is effective in reducing airborne infection risk *P*.

Fieldwork using CO_2_ sensors as a countermeasure against airborne transmission has been conducted worldwide. Wiryasaputra et al. [21] conducted a systematic review of studies that monitored indoor environments, particularly CO_2_, to reduce the risk of COVID-19 infection. According to a systematic review of 19 articles in urban spaces, CO_2_ is the primary reference for the spread of novel coronaviruses in buildings [22]. Studies have been conducted to visualize areas at high risk of COVID-19 infection using the CO_2_ tracer gas method [23–27]. In South Africa, where TB is widely prevalent, CO_2_ sensors have been installed in public buildings, and the application of Eq (1) to estimate the risk of TB transmission has confirmed that the chances of transmission in schools is relatively large [28, 29]. Additionally, attempts have been made in South Africa to utilize portable CO_2_ sensors with a built-in global positioning system (GPS) [30] and combine them with a geographic information system to visualize the risk of TB transmission on a map [31]. However, to the best of our knowledge, no attempts have been made to employ citizens with mobile CO_2_ sensors to measure the adequacy of ventilation in developed countries or metropolitan areas during the COVID-19 pandemic.

Therefore, this study aimed to demonstrate the feasibility of visualizing the risk of airborne infection with mobile citizen sensing using a Pocket CO_2_ Logger to identify locations with poor ventilation in the Tokyo area, the largest megacity in the world.

## Materials and methods

### Pocket CO_2_ Logger

**Fig 1:**
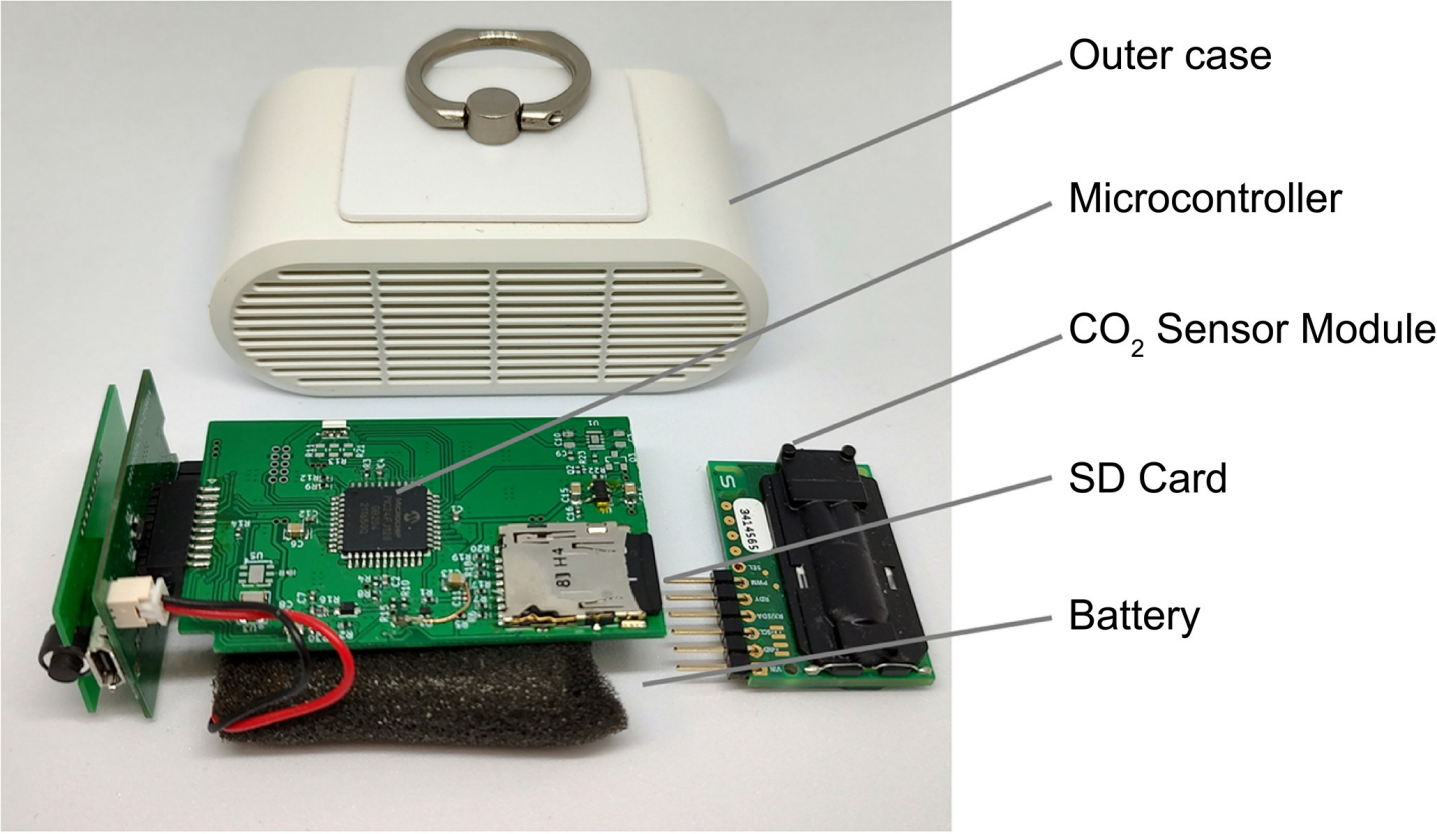
Pocket CO_2_ Logger.

The Pocket CO_2_ Logger (Fig 1), which we developed originally, measures 76.5 mm wide, 30 mm height, and 43 mm in depth and weighs 40 g for easy portability. The device is equipped with a nondispersive infrared CO_2_ sensor module, SCD30 (Sensirion AG., Stäfa, Switzerland), a lithium polymer battery, a low-power microcontroller in deep-sleep mode (PIC24FJ128GB204, Microchip Technology, Arizona), a real-time clock (RX8900CE, Seiko Epson, Tokyo, Japan), and a 1 GB SD card.

By default setting, intermittent operation every 10 min and a 10 s pre-measurement warm-up ensure continuous operation for at least one week, since the sensor consumes no power except while measuring. These variables for intermittent operation and warm-up time can be changed arbitrarily in the configuration file on the SD card. The built-in real-time clock has low power consumption and long-term operation by virtue of its deep-sleeping qualities outside the measurement time.

Yasuda et al. [30] have developed the Portable CO_2_ Measurement Device with a handle and powered by six AA cell batteries, although it is considered excessively large and not portable enough for participants to carry with them at all times. Wood et al. [31] developed a true portable CO_2_ logger that can be carried by participants for TB prevention in Cape Town, South Africa; however, the Pocket CO_2_ logger is 34% smaller in volume and has 3.5 times longer continuous operating time than Wood’s logger. Ishigaki and Kitamura et al. [25–27] deployed TR-76Ui sensors (T&D Corporation, Nagano, Japan) for mobile CO_2_ logging to estimate the risk of airborne transmission of COVID-19; however, the Pocket CO_2_ logger is 66% smaller in volume and 92% lighter in weight than TR-76Ui. The USB connector can be used for both charging and data transfer in accordance with the USB mass storage mode. The measured results were recorded on the SD card in a comma-separated value (CSV) format as log file(s), with one file generated per day with the name of that date. The log files could subsequently be transferred to a computer via USB connection.

Nondispersive infrared sensing is a method of estimating CO_2_ concentration by measuring the attenuation of the absorption wavelength (4.26 μm) of CO_2_ molecules using a photodiode. The internal sensor module (SCD30) also contained temperature and humidity sensors that were used to compensate for the CO_2_ concentration measurements. The CO_2_ measurement range of SCD30 is 400–10,000 ppm, with a measurement accuracy of ±30 ppm, typical relative humidity accuracy of 3% RH, and operating relative humidity range of 0–95% RH; hence, it is unlikely to be significantly inhibited in everyday temperature and humidity environments.

### Participants

With the cooperation of a local restaurant in Tokyo, Japan, we have requested 11 customers to participate in the experiment. Those who consented were lent a Pocket CO_2_ Logger and asked to carry it with them at all times for the intended one-week experimental period, except when directly exposed to water, such as when taking a bath. The Pocket CO_2_ Logger can operate for more than a week without requiring a recharge and could be carried at all times.

The participants were instructed to carry the Pocket CO_2_ Logger in one of three possible ways: hang it around their neck with a strap, attach it to a carabiner, or tie it to a bag or pouch. During the experiment, the participants recorded in the logger the location and time of their stay. This allowed the continuous collection of data on the participant’s locations and CO_2_ concentrations. However, for privacy protection reasons, we did not collect any GPS or location information, nor record the names or contact information of the participants, except for sex and history of COVID-19 infection. Thus, the data of the measurement results were not linked to any specific individual.

This study was approved by the Ethics Committee of the University of Electro-Communications, Chofugaoka, Chofu, Tokyo, Japan (approval number: 21006 and 21006 [2]). The need for consent was waived by the ethics committee. Participants were provided with written information about the purpose of the experiment and contact information for questions. The participants did not include minors. The recruitment period for this study started June 2, 2021and ended March 31, 2022.

### Data analysis

Based on the data of the participant’s identification (ID), CO_2_ concentration [ppm], and location listed in the ledger, we performed an analysis using JMP Pro 16.2.0 (SAS Institute, North Carolina) with a generalized linear mixed model (GLMM). The objective variable was set to the logarithm of CO_2_ concentration, participant ID was assigned as the random effect, and location was assigned as the fixed effect. Because the description of the location was expected to vary between participants, it was screened in advance and set as a factor after roughly classifying the genres as follows: cinema, gyms, halls, homes, hospitals, pubs, restaurants, universities, stores, transportation, workplaces, other indoors, and other outgoings.

The data may have contained participant-specific biases owing to the different carrying methods used by each participant. Therefore, as a preliminary analysis, we estimated the covariance parameter of the random effect of participant ID, checked the validity of conducting an analysis incorporating the random effect, determined whether individual differences were significant based on the Wald *p*-values (double-sided), and confirmed that significant individual differences were found.

In parallel with the above analysis, quantile plots and histograms were generated for the residuals and random effects of the estimated GLMM, respectively, to validate the use of the GLMM and the normality of the variable. A priori determination of the necessary sample size or post-hoc power analysis was not performed as this was a pilot study.

## Results

**Fig 2:**
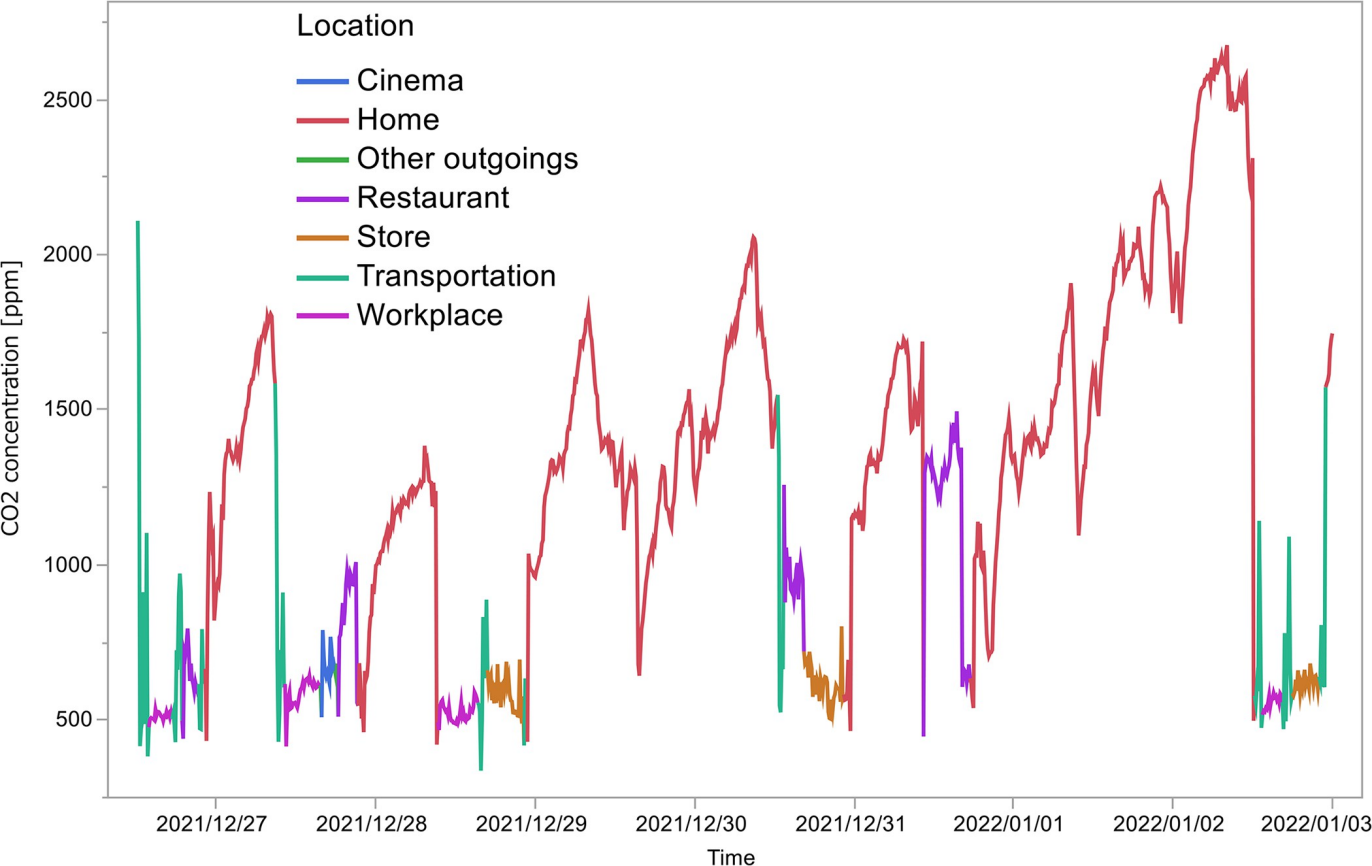
Trend of CO_2_ concentration for one participant. Measurements are color-coded for each location and connected by a line graph.

An average of 8 days of data per participant was collected from the 11 participants (seven males and four females, none with a history of COVID-19 infection) from December 8, 2021 to January 14, 2022, resulting in a total of 12,253 records. The start date of the experiment varied between participants. As an example, Fig 2 plots the CO_2_ concentration for one participant (ID: C05), color-coded according to location. It shows the trend of CO_2_ concentration changing at different locations; at home, the CO_2_ concentration increased monotonically with time spent at home, whereas at a restaurant and store, the CO_2_ concentration was steady at a lower level than at home.

The data collected from all participants were tabulated and the number of records per location in descending order was as follows: home, 8081 records (66.0%); workplace, 1963 records (16.0%); other outgoings, 866 records (7.1%); transportation, 368 records (3.0%); restaurant, 343 records (2.8%); hall, 191 records (1.6%); store, 186 records (1.5%); gym, 75 records (0.6%); pub, 69 records (0.6%); other indoors, 48 records (0.4%); hospital, 39 records (0.3%); cinema, 12 records (0.1%); and university, 12 records (0.1%).

A Wald *p*-value of 0.0342 for participant ID in the covariance parameter estimates of the variant effects showed significant individual differences, confirming the validity of setting participant ID as a random effect in the GLMM. S1 Fig in S1 File shows a box-and-whisker diagram of the variation in CO_2_ concentration for each participant over the study period.

The histogram and quantile plot of the estimated GLMM residuals are shown in S2 Fig in S1 File, and the histogram and quantile plot of random effects indicating individual differences are shown in S3 Fig in S1 File. In both cases, the distributions were considered approximately normal; therefore, the application of GLMMs was considered reasonable.

Table 1 summarizes the parameter estimators for the fixed effects. These estimators show the interaction of each factor, sorted in ascending order by the estimator. The factors indicating locations that significantly (*p* < 0.0001) contributed to CO_2_ concentrations were, in ascending order, workplaces, halls, homes, gyms, other indoors, pubs, other outgoings, stores, and universities. Conversely, the results for hospitals, transportation, restaurants, and cinemas were not significant.

**Table 1:** Parameter estimators for fixed effect: Sorted in ascending order by estimator.

Factor	Estimator	*t*-value	*p*-value	95% CI lower	95% CI upper
Workplace ^#^	6.836653	69.55	<0.0001*	6.615868	7.057439
Hall	0.517114	16.54	<0.0001*	0.455837	0.578392
Home	0.391545	23.91	<0.0001*	0.359439	0.42365
Gym	0.30746	6.77	<0.0001*	0.218488	0.396431
Other indoors	0.256294	4.56	<0.0001*	0.14608	0.366508
Pub	0.2545	5.33	<0.0001*	0.160934	0.348066
Hospital	0.097899	1.6	0.1085	-0.021669	0.217466
Transportation	-0.010187	-0.41	0.6829	-0.059063	0.03869
Restaurant	-0.027043	-1.07	0.2836	-0.07648	0.022395
Other outgoings	-0.113492	-5.24	<0.0001*	-0.155941	-0.071044
Cinema	-0.269897	-2.52	0.0118	-0.480056	-0.059739
Store	-0.302479	-9.65	<0.0001*	-0.36395	-0.241009
University	-0.960095	-8.93	<0.0001*	-1.170827	-0.749363

CI: Confidence interval

# indicates intercept.

* indicates a significant difference (<0.05).

**Fig 3:**
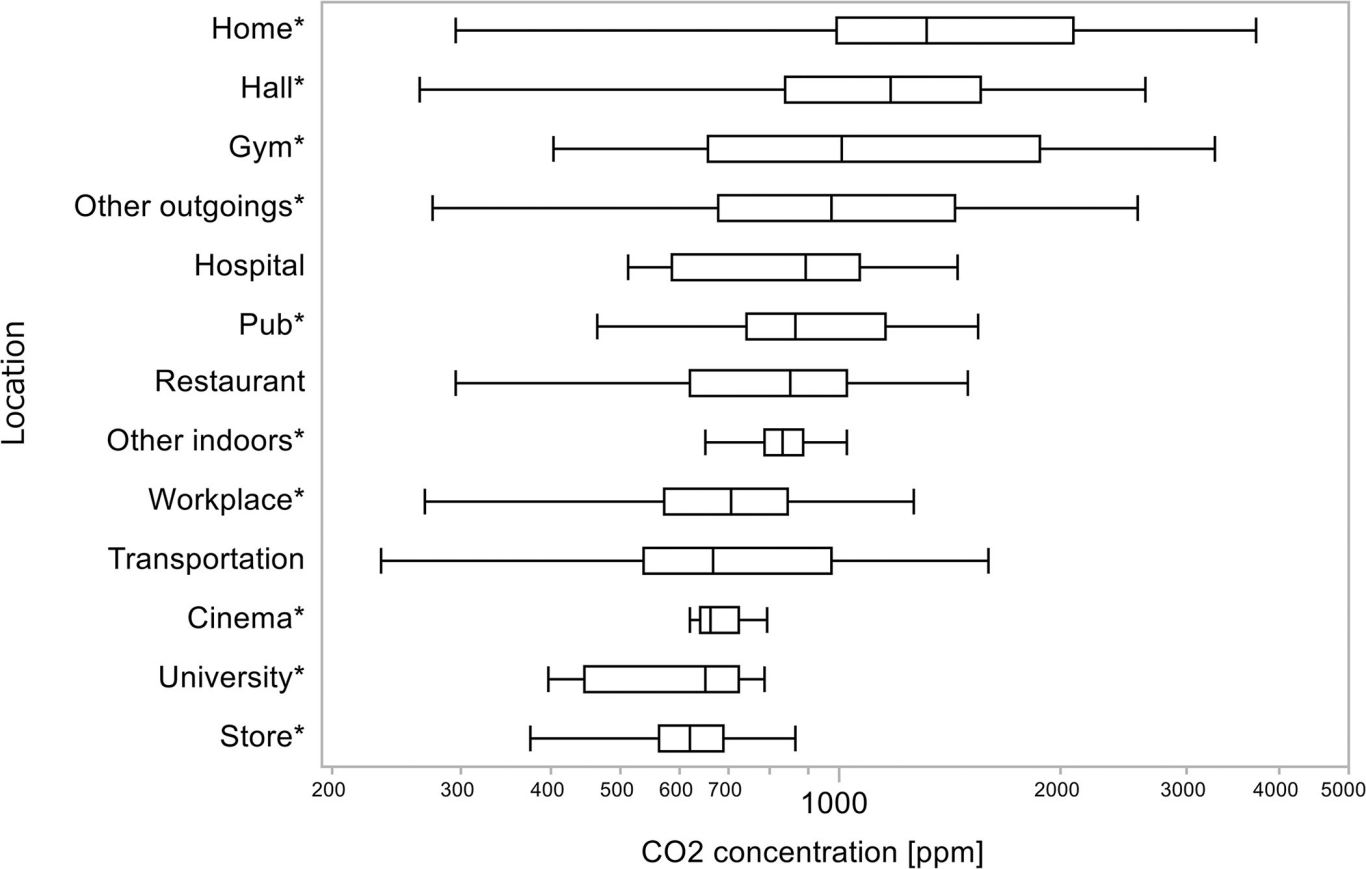
Box-and-whisker plot of CO_2_ concentration by location. The locations are sorted in descending order of average values. Asterisk (*) indicates that the factor contributed significantly (*p* < 0.05) to the CO_2_ concentration.

Fig 3 shows a box-and-whisker plot of the CO_2_ concentrations plotted by location. Factors that contributed significantly to the CO_2_ concentration obtained from the parameter estimates above are marked with an asterisk (*).

## Discussion

Of the locations considered relatively poorly ventilated, with a median CO_2_ concentration exceeding 1,000 ppm in all measurements (Fig 3), those contributing significantly (*p* < 0.0001) (Table 1) were: homes (1,316 ppm), halls (1,173 ppm), gyms (1,005 ppm), and other outgoings (972 ppm). Conversely, the locations with relatively good ventilation, with a median value <1,000 ppm, and continued to significantly contribute to CO_2_ concentrations were: pubs (864 ppm), other indoors (835 ppm), workplaces (705 ppm), cinemas (663 ppm), universities (652 ppm), and stores (620 ppm).

Our study highlights homes as being poorly ventilated enclosed spaces, despite a previous study examining the risk of TB transmission in South Africa pointing to schools and public facilities [32]. Although Japan’s Building Standards Act requires 0.5 times/h ventilation to prevent sick house syndrome, this alone is not enough to maintain CO_2_ concentrations <1,000 ppm. A study conducted in China by the World Health Organization [33] indicated that 80% of COVID-19 outbreaks could be attributed to household infections. The actual risk of secondary infection at home is 12.1% in Japan (Okinawa), 4.6% in Taiwan, 10.5% in the USA, 11.8% in Korea, and 17.2% in China [33–38]. These findings of a relatively high risk of infection at home are consistent with the results of this study.

Outbreaks due to airborne transmission during choruses have been confirmed in the U.S. and Australia [13, 39]. Clusters also occurred during yoga sessions in poorly ventilated gyms in South Korea [40]. In developed countries, halls and gyms are often built in dense residential areas; hence, windows may not be opened to prevent sound pollution and promote privacy. Such urban-specific environments are thought to increase the CO_2_ concentrations in homes, halls, and gyms.

For other outgoings, a relatively large amount of data was collected– 866 records (7.1%)–but the classification was rather ambiguous and included incidences when participants walked outdoors or in other unclassified buildings. Future studies should analyze data in a more detail by specifically stating the type of building.

For pubs, the median CO_2_ concentration was <1,000 ppm, which was relatively low. The Tokyo Metropolitan Government issued multiple emergency declarations requiring pubs to reduce business hours or refrain from operating in cooperation with the police and fire department, with business owners who cooperated receiving financial subsidies [41]. To deter airborne transmission of infection, concurrent measures should be considered for high-risk locations such as homes, halls, and gyms. For example, the Kyoto Prefecture installed CO_2_ sensors free of charge in 2,836 restaurants in the prefecture, and based on the data on collected, made individual visits and provided guidance on specific measures to improve ventilation [42]. To deter infections in family members during home care, it may be effective to conduct surveillance based on CO_2_ concentrations at home.

In contrast, CO_2_ concentrations were relatively low in workplaces and stores. This could be attributed to the Act on Maintenance of Sanitation in Buildings (enforced in 1970), which mandates CO_2_ concentrations of 1,000 ppm or less in entertainment venues, large-scale stores, offices, and school, along with periodic inspections.

The data for "other indoors, cinemas, and universities" is not sufficient because only one participant visited these places. This may be because the activities of the participants were restricted because of the Japanese government’s request for a voluntary curfew.

Notably, certain hidden variables, including the number of people staying, metabolic rate, air area of the space where the participants stayed, and ventilation frequency, were not addressed in this study, indicating its weakness. The CO_2_ concentration fluctuates and stabilizes with these variables [43]. It would be possible to adopt the CO_2_ concentration as a reliable risk proxy by knowing what the steady-state CO_2_ concentration is in the space where the participant temporarily stays. For this purpose, installing fixed CO_2_ sensors and human detection cameras on the space side are necessary.

This study had several limitations. First, it did not capture the demographic characteristics of the participants (excluding sex and history of COVID-19); hence, we were unable to analyze characteristics according to age group or employment status. Second, since the location information was identified by self-report, it may contain errors or mistakes. Future research should link the indoor and outdoor location estimation functions of smartphones using GPS and Wi-Fi to automatically collect location information. If smartphones and sensors can automatically synchronize via Bluetooth and periodically collect data in the Cloud, the workload of the participants can be significantly reduced, resulting in large-scale, long-term monitoring with a larger number of individuals. This can contribute to field epidemiology and administrative decision-making.

In this study, the CO_2_ concentration was classified as high or low based on a cut-off of 1,000 ppm; however, for future social implementation, a method to adaptively calculate the excess CO_2_ threshold based on the number of occupants, community prevalence, and activity level should be adopted [44].

## Conclusions

Using our newly developed, compact, and lightweight Pocket CO_2_ Logger, we found that homes, halls, and gyms, were relatively poorly ventilated and significantly (*p* < 0.0001) contributed to elevated CO_2_ concentrations, with average values >1,000 ppm. As several cases of outbreaks due to airborne transmission in these locations have been reported in previous studies, it can be inferred that they have a relatively high risk of transmission.

In contrast, workplaces and stores had significantly lower (*p* < 0.0001) CO_2_ concentrations. This may be due to the ventilation regulations under Japan’s Act on the Maintenance of Sanitation in Buildings.

Our results may be used to make policy recommendations for infection control, such as human flow restrictions and ventilation measures, in locations identified as relatively high-risk. In the future, we plan to conduct a large-scale survey and the sensor may be linked to a smartphone to automatically collect indoor and outdoor location information to reduce the workload on the participant.

## Supporting information

S1 File (DOCX)
