# Supplementary material for: Monitoring the ventilation of living spaces to assess the risk of airborne transmission of infection using a novel Pocket CO2 Logger to track carbon dioxide concentrations in Tokyo
Source: PLoS One. 2024 May 23;19(5):e0303790. doi: 10.1371/journal.pone.0303790 (PMC11115307; doi:10.1371/journal.pone.0303790)
Supplement: S1 File — (DOCX) [file pone.0303790.s001.docx]

**Supporting Information**


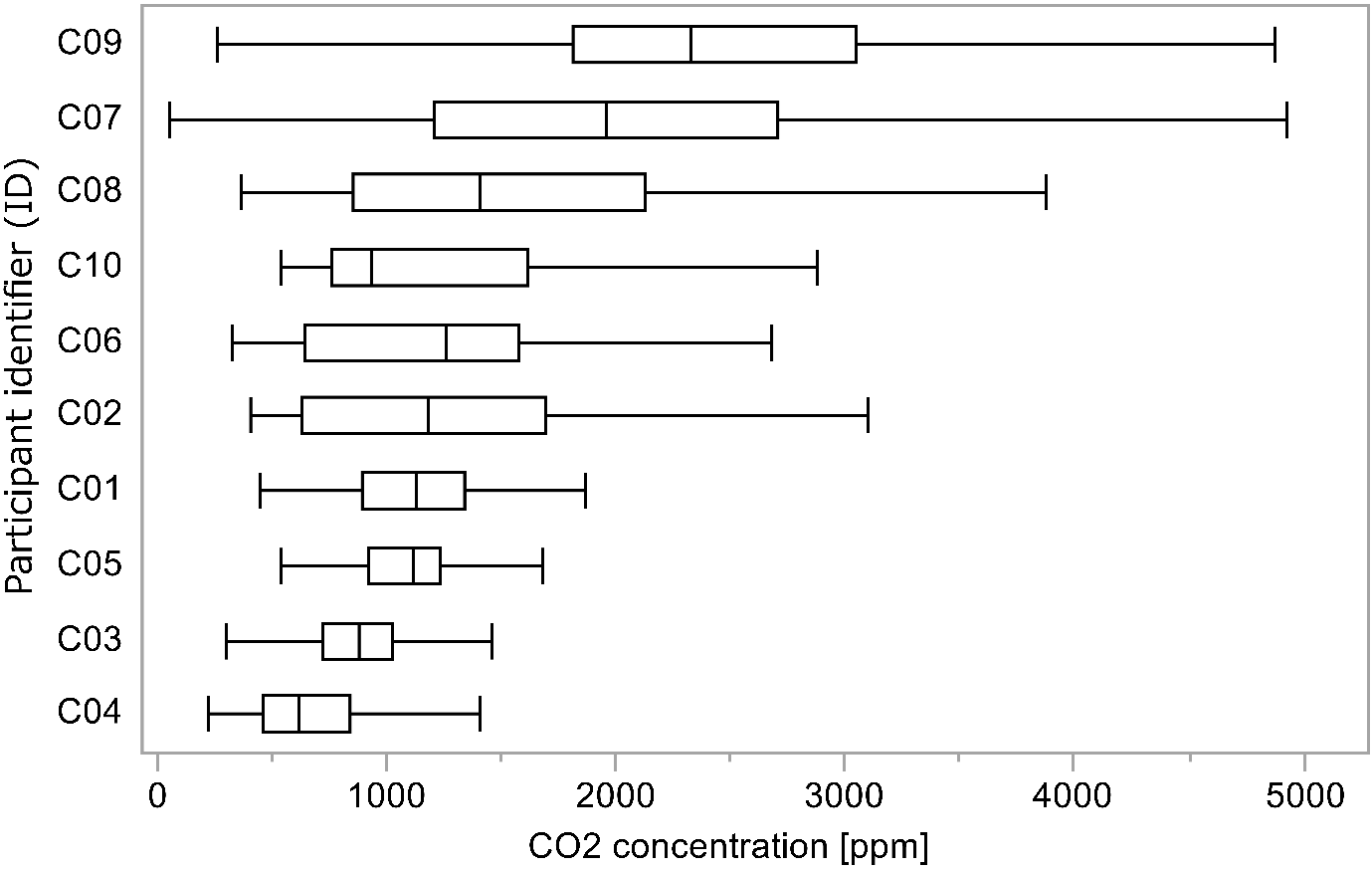


**S1 Figure. Box-and-whisker plot of CO_2_ concentrations for each participant during the study period**. CO_2_, carbon dioxide


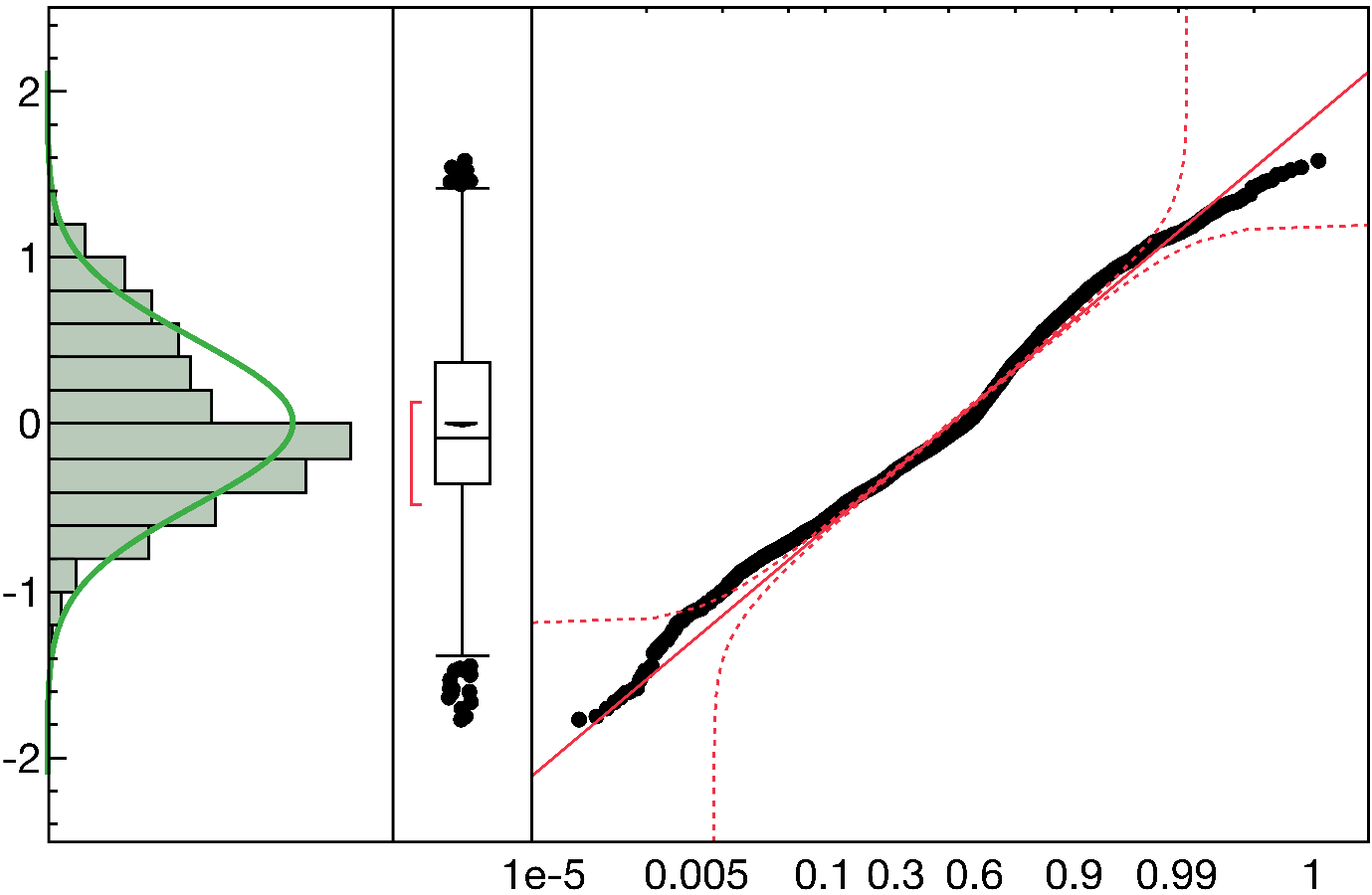


**S2 Figure. The histogram and quantile plot of the GLMM residuals CO_2_**. CO_2_, carbon dioxide; GLMM, generalized linear mixed model


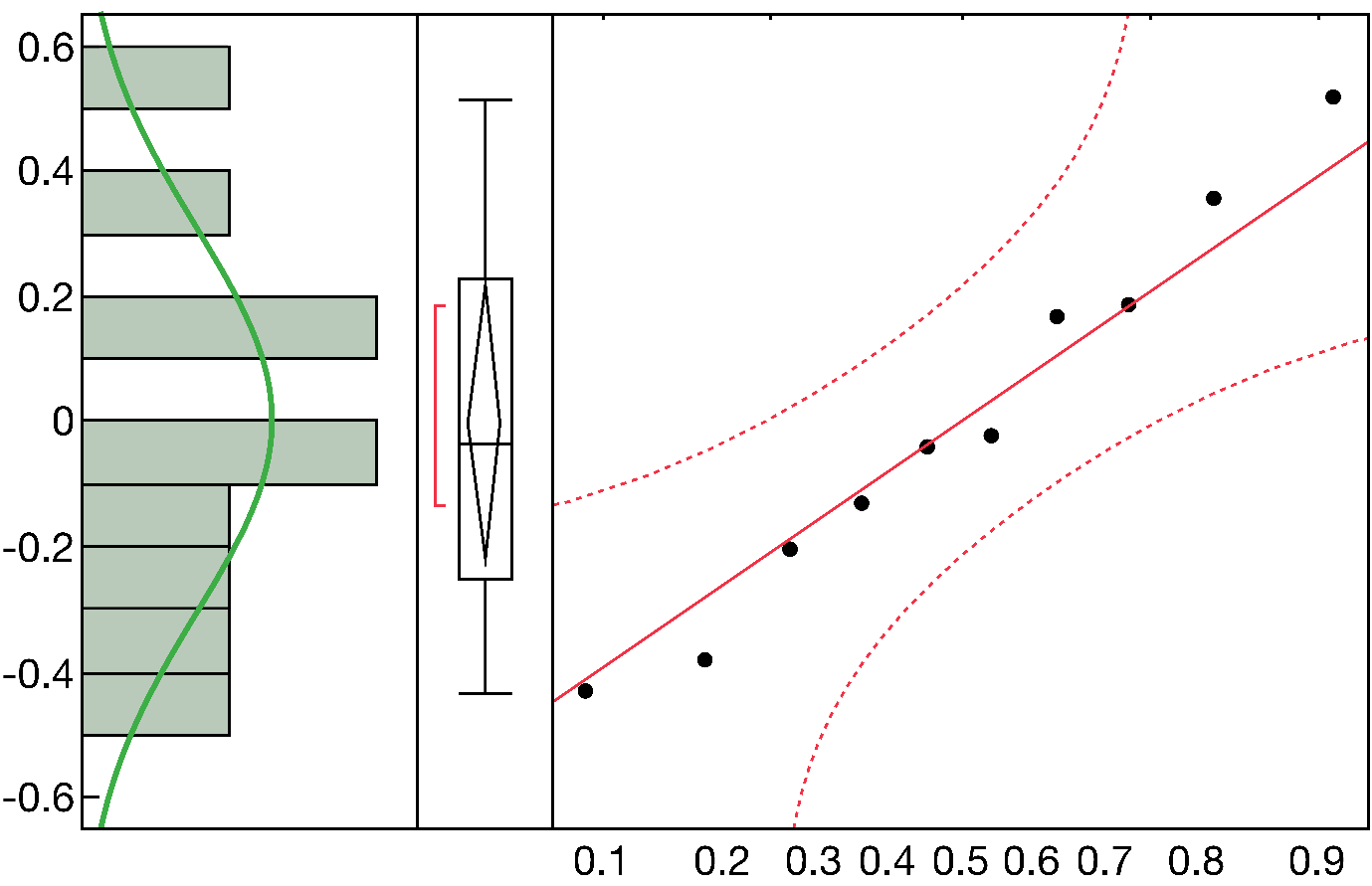


**S3 Figure. The histogram and quantile plot of the random effects of the GLMM**. CO_2_, carbon dioxide; GLMM, generalized linear mixed model
